# Supported online self-management versus care as usual for symptoms of fatigue, pain and urgency/incontinence in adults with inflammatory bowel disease (IBD-BOOST): study protocol for a randomised controlled trial—an update to the published protocol

**DOI:** 10.1186/s13063-023-07095-5

**Published:** 2023-02-03

**Authors:** Christine Norton

**Affiliations:** King’s College London, London, UK

This document reports an update to the protocol of the IBD-BOOST study published in Trials [1]. The statistical analysis of this study has been described in that publication. We have updated our protocol as follows (changes in italics):

**Primary end points:** *We have clarified how exactly our two primary outcomes at 6 months are used. Primary outcomes: IBD quality of life (UK-IBDQ scale) and global rating of symptom relief scale at 6 months as multiple primary outcomes with multiplicity adjustment (rather than as co-primary outcomes: see* *https://www.ema.europa.eu/en/documents/scientific-guideline/draft-guideline-multiplicity-issues-clinical-trials_en.pdf**).*

**Sample size** ***(increased from 680 to 740):*** *An original sample size calculation estimated that 680 participants would need to be recruited to the trial and the original funding and ethics approval was on this basis. However, during the study, the COVID-19 pandemic made our anticipated recruitment of 30 facilitators from 20 NHS sites impossible. During team discussions, it also became evident that we had not made appropriate statistical adjustments for having two primary end points. The sample size calculation was therefore adjusted before the end of recruitment as follows:*

*A minimum of 740 participants will be randomised, approximately 370 to each group. This allows the MCID difference to be detected with 86.4% power at a 2.5% significance level. It is anticipated that 16 facilitators are participating in the trial. Taking account of a facilitator effect (assuming a facilitator intraclass correlation of 0.04) in the intervention arm, 352 participants are required in each study arm to achieve 86.4% power (21 participants per facilitator). The sample size is decreased by a deflation factor of 0.84 assuming that baseline values of the outcome measure are predictive of post-treatment values (correlation 0.4) and inflated to account for 20% loss to follow-up resulting in the final recruitment target of 740.*

*Adjustment for correlation between baseline and follow-up values of the primary outcomes is based on a median correlation of a QoL measure with 6 months post-randomisation outcome of 0.5 with a lower IQR bound of 0.41. Being conservative, a correlation of 0.40 is assumed.*

**Blinding:** We have clarified our statement on blinding: *The trial steering committee, CI, health economics and statistics teams are blinded, that is, will not see results broken down by treatment arm during the trial or any time prior to analysis plans being signed off. Final analysis will occur once all follow-up data is collected, the final statistical analysis plan has been signed off and data cleaning has occurred. There is no DMEC for this trial, which was assessed as low risk by our clinical trials unit, with the steering group acting in the capacity (and reviewing blinded safety data at their meetings). Only if there were concerns would the necessity for unblinding be considered. There are no plans to routinely review interim unblinded outcome and safety data.*

**Statistical analysis:** We have made an adjustment to accommodate multiple comparisons: *Analyses will be performed according to intention-to-treat; all patients with a recorded outcome will be included in the analysis and analysed according to the treatment to which they were randomised. Summary statistics by group, treatment effects, 95% confidence intervals, and p-values will be presented for primary and secondary outcomes, and process measures. Baseline demographics and outcomes, type of IBD classification, Rome IV IBS and all other follow up data for the two groups will be summarised by treatment group using descriptive statistics, but not subjected to statistical testing.*

*The two primary outcomes UK-IBDQ and global rating of symptom relief will be compared between the two arms at 6 months after randomisation to assess treatment effectiveness. The significance level for hypothesis testing of the primary outcomes will be Bonferroni adjusted to alpha = 0.025 to allow for multiple comparisons. There will be no multiplicity adjustment of the significance level for hypothesis testing of the pre-specified secondary outcome measures. The number of comparisons will be duly taken into account when interpreting the results and in particular claims for secondary outcomes will be cautious if no difference in the primary outcomes is detected. The primary outcomes will be analysed using mixed model repeated measures analysis with restricted maximum likelihood estimation and an unstructured covariance matrix for residuals. The following covariates will be included in the model as fixed effects: baseline value of outcome measure, stratification factors, fatigue, pain and incontinence at baseline, age and gender. Facilitators will be added as a random effect in the intervention arm only.*

The study protocol is otherwise unchanged. *Because of delays caused by the COVID-19 pandemic, we have extended our recruitment period until 31.07.22.* These changes have been approved by our ethics committee, updated in our International Trials Registration document (ISRCTN71618461) and implemented prior to completion of recruitment and prior to finalising our statistical analysis plan.

## Data Availability

Not applicable.
